# Identification of protein–protein interaction bridges for multiple sclerosis

**DOI:** 10.1093/bioinformatics/btad175

**Published:** 2023-04-05

**Authors:** Gözde Yazıcı, Burcu Kurt Vatandaslar, Ilknur Aydin Canturk, Fatmagul I Aydinli, Ozge Arici Duz, Emre Karakoc, Bilal E Kerman, Can Alkan

**Affiliations:** Department of Computer Engineering, Bilkent University, Ankara, Turkey; Research Institute for Health Sciences and Technologies (SABITA), Istanbul Medipol University, Istanbul, Turkey; Graduate School of Health Sciences, Department of Neuroscience, Istanbul Medipol University, Istanbul, Turkey; Goztepe Prof. Dr. Suleyman Yalcin City Hospital, Istanbul, Turkey; Research Institute for Health Sciences and Technologies (SABITA), Istanbul Medipol University, Istanbul, Turkey; Department of Medical Biology, School of Medicine, Nisantasi University, Istanbul, Turkey; Faculty of Medicine, Department of Neurology, Istanbul Medipol University, Istanbul, Turkey; Wellcome Sanger Institute, Hinxton, United Kingdom; Research Institute for Health Sciences and Technologies (SABITA), Istanbul Medipol University, Istanbul, Turkey; Department of Medicine, Keck School of Medicine, University of Southern California, Los Angeles, California, United States; Department of Computer Engineering, Bilkent University, Ankara, Turkey

## Abstract

**Motivation:**

Identifying and prioritizing disease-related proteins is an important scientific problem to develop proper treatments. Network science has become an important discipline to prioritize such proteins. Multiple sclerosis, an autoimmune disease for which there is still no cure, is characterized by a damaging process called demyelination. Demyelination is the destruction of myelin, a structure facilitating fast transmission of neuron impulses, and oligodendrocytes, the cells producing myelin, by immune cells. Identifying the proteins that have special features on the network formed by the proteins of oligodendrocyte and immune cells can reveal useful information about the disease.

**Results:**

We investigated the most significant protein pairs that we define as bridges among the proteins providing the interaction between the two cells in demyelination, in the networks formed by the oligodendrocyte and each type of two immune cells (i.e. macrophage and T-cell) using network analysis techniques and integer programming. The reason, we investigated these specialized hubs was that a problem related to these proteins might impose a bigger damage in the system. We showed that 61%–100% of the proteins our model detected, depending on parameterization, have already been associated with multiple sclerosis. We further observed the mRNA expression levels of several proteins we prioritized significantly decreased in human peripheral blood mononuclear cells of multiple sclerosis patients. We therefore present a model, BriFin, which can be used for analyzing processes where interactions of two cell types play an important role.

**Availability and implementation:**

BriFin is available at https://github.com/BilkentCompGen/brifin.

## 1 Introduction

Oligodendrocytes, which are specialized glial cells of the central nervous system, surround axons with their plasma membrane and form the myelin sheath required for the proper functioning of the vertebrate nervous system. By virtue of the insulation supplied by myelin sheath, action potentials propagate faster along myelinated axons than on non-myelinated axons (Hartline and Colman 2007). In addition, oligodendrocytes provide metabolic support to neurons promoting long-term neuronal survival and function (Fünfschilling et al. 2012, Saab and Nave 2017). Loss of myelin (demyelination) triggers a neurodegeneration cascade causing the disruption of electrical signal transmission, deprivation of oligodendroglial support, and thus further damage to neurons. Demyelination occurs in several neurological diseases, one of the most prevalent being multiple sclerosis (MS) (Traka et al. 2016, Kipp 2020).

Affecting over 2 million people, MS is a chronic, inflammatory, and neurodegenerative disease in which demyelination is observed in the various regions of the brain and spinal cord (SC) (Garg and Smith 2015, Stadelmann et al. 2019, Walton et al. 2020). The etiology of the disease is thought to be immune dysregulation resulting from the interactions of genetic and environmental factors (Olsson et al. 2017). Abnormally activated immune cells attack and damage myelin. Due to the impairments of interneuronal communication and signal transmission, physical and cognitive symptoms are observed in MS patients. Clinically, the majority of patients exhibit a relapsing-remitting phenotype of MS (RRMS) characterized by episodes of reversible neurological attacks followed by total or local recovery. In time, in patients diagnosed with RRMS, neurologic deficits become permanent, and secondary progressive MS develops (Lucchinetti et al. 2008, Rajendran et al. 2021).

Inflammation, axonal loss, and oligodendrocyte and neuronal cell death are the hallmarks of MS lesions. Lymphocytes, microglia, and macrophages are the main immune cell types that contribute to inflammation in the lesion areas (Garg and Smith 2015). Therefore, it is important to reveal the cell-to-cell interaction mechanisms between oligodendrocytes and immune cells to understand MS disease mechanisms.

In the last decade, network medicine has become an important discipline to analyze disease etiology. Network medicine (Barabási et al. 2011) focuses on analyzing biological networks with a holistic view instead of analyzing a single gene or a mechanism to find mechanisms or important actors of diseases. Diverse biological networks are analyzed for different purposes. In this study, we focus on protein–protein interaction (PPI) networks formed between two cells, the target and the perpetrators of demyelination since PPI networks may provide the necessary information to identify the key interacting proteins that are involved in demyelination. For a PPI network, the question to which network medicine seeks an answer is whether the disease-associated proteins carry special attributes on the network. One of the current research directions for this question is to predict new disease-associated proteins using the existing knowledge on disease-associated proteins and machine learning-based algorithms (Tieri et al. 2019). Several studies try to find associations between genes or proteins and diseases through genomic and transcriptomic data that is included in a gene expression or a protein interaction network, and previously identified disease-associated genes or proteins. For example, HIT’nDRIVE, proposed by Shrestha et al. (2017) is a comprehensive computational method that integrates transcriptomic and genomic data and it aims to find the smallest set of patient-specific altered genes on the network that can cause transcriptional perturbations.

An alternative network analysis strategy is detecting hubs, which are the nodes that are few in number, yet have the highest degrees in the network. Hub proteins are not necessarily disease-associated proteins; however, the reason why they should be investigated is the fact that they are involved in many interactions in the network. Therefore, a change in their interactions results in relatively more significant biological alterations. In this study, we detected the hubs in the PPI networks formed within and between the cells that play a role in demyelination for further biological research. Here, the hubs, we refer to are “specialized” hubs, which connect two cells and we call ‘bridges’ in the remainder of this article. Since we analyzed a network by combining two different networks through intercellular PPIs, we identified these bridges as the protein pairs that had the highest ‘intracellular importance scores’ (IIS) (see Section 2), and that are involved in the highest number of the intercellular interactions. In this study, our aim is to detect bridges on the demyelination PPI network that might likely play a role in the development of MS.

In contrast to analyzing a PPI network generated from healthy cells, analyzing the PPI network generated from disease-carrying cells is also a promising way to understand disease etiology. For example, Yurduseven et al. (2022) identified MS biomarkers by performing interactome analysis using an MS-specific brain PPI network that they constructed using transcriptome data. Yurduseven et al. (2022) analyzed cell-type-specific and cell-to-cell bridges by considering the degrees of the proteins on the MS-specific brain PPI networks.

Here, we introduce Bridge Finder (BriFin) to detect the bridges in a cell-to-cell protein interaction network that consists of both inter- and intracellular interactions between two cell types. Using BriFin, we identify proteins that may be associated with MS, specifically through playing key roles in immune cell–oligodendrocyte communications.

Revealing the proteins that take part in the disease mechanism is not an easy task due to the difficulty of obtaining biological samples from the brain and SC where MS involvements are observed. Therefore, we evaluated BriFin by investigating some MS-associated potential biomarkers in easily available peripheral blood mononuclear cell (PBMC) samples, which may contribute to the understanding of the disease mechanism, as well as being used in the diagnosis and follow-up of the disease. Among the proteins with the highest scores, we selected four that are likely important in MS pathogenesis and verified the expression levels of the genes that code these proteins in PBMCs of MS patients using quantitative real-time PCR (qRT-PCR). We showed that the expression levels of two out of four genes that code the suspect proteins were significantly decreased in MS patients, which suggests disrupted downstream networks. In addition, we showed that the rate of the MS-associated proteins BriFin detected varies between 61% and 100% depending on the parameterization regarding the desired prioritization level. Furthermore, we showed that the MS-associated protein detection rate of BriFin increases by the ascending prioritization level.

## 2 Materials and methods

### 2.1 Dataset and network construction

**Figure 1.**
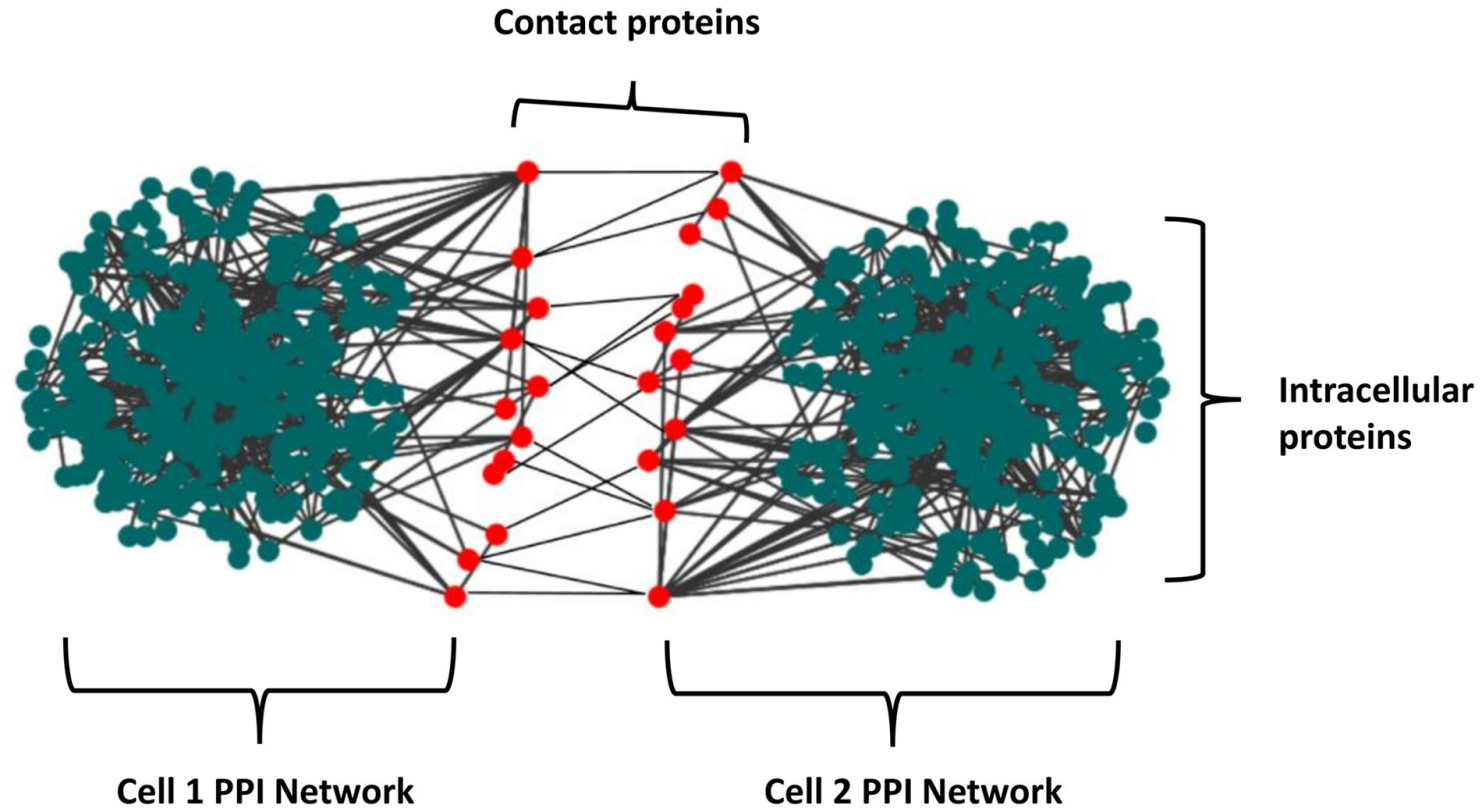
Constructed network and problem terms.

Recent advances in sequencing technologies and increasing biological data available in public databases enable us to better model and understand cell-to-cell interactions and protein networks (Armingol et al. 2021). In this study, we obtained the proteome data for the three different cell types from several articles (Dupont et al. 2004, Slomianny et al. 2006, Ishii et al. 2009, Raposo et al. 2011, de Monasterio-Schrader et al. 2012, Lichtenfels et al. 2012, Iwata et al. 2013, Eligini et al. 2015, Graessel et al. 2015, Mitchell et al. 2015, Pagani et al. 2015, Joshi et al. 2019) and publicly available databases [UniProt (Bairoch et al. 2005) and The Human Protein Atlas (Uhlén et al. 2015)]. Next, we downloaded the PPI network data for the intracellular interactions from the IntAct Molecular Interaction Database (Hermjakob et al. 2004). We identified the probable contact proteins (membrane and secreted proteins) and the intercellular interactions for each cell using the data of ligand–receptor pairs from the cell-to-cell communication databases [CellTalkDB (Shao et al. 2021) and BaderLab ligand–receptor interaction set (https://baderlab.org/CellCellInteractions)], since cell-to-cell interactions generally involve ligand–receptor pairs. These databases that include curated data from diverse resources aim at presenting the protein pairs that involve in human cell-to-cell protein interactions. After identifying the contact proteins, we removed the unconnected nodes from the network and finally calculated PageRank and Betweenness Centrality scores for all nodes (i.e. proteins) using the Gephi network analysis tool (Bastian et al. 2009). All edges are undirected in the constructed networks. In Fig. 1, we present a visual of the constructed network and the problem terms.

### 2.2 Network pruning and IIS

We initially compiled data for two large interacting PPI networks, namely oligodendrocyte–macrophage and oligodendrocyte–T-cell networks, where all intra- and intercellular interactions are included. However, both of these PPI networks were very large: the oligodendrocyte–macrophage network contained 3574 nodes and 33 399 edges, while the oligodendrocyte–T-cell network contained 11 813 nodes and 152 396 edges. To reduce the problem size and to be able to solve it efficiently, we assigned an ‘intracellular importance score (IIS)’ to each contact protein expressing its importance for the intracellular network of the cell it belongs to. To do this, we first determined each contact protein’s direct and indirect interactors, and their distances using a breadth first search strategy. We evaluated the importance of the individual proteins in the intracellular network based on their PageRank centrality values. We used the default parameters in Gephi to calculate the PageRank scores, which are 0.85 for damping factor, and 0.001 for epsilon.

There are various strategies to assess the importance of a node of the network, such as using one of the centrality metrics, using a combination of them or designing a new problem-specific metric. As previously mentioned, we chose to use PageRank centrality to measure the importance of an individual node. We assumed that all intracellular biological interactions are equally important, and we aim to identify to what extent an intracellular protein is involved in these interactions. We therefore use PageRank that assumes a random surfer on the network, and measures the likelihood of being visited of a node.

Combining the connection and score information, we assigned scores for each contact protein using Equation (1). Similarly, Al-Fatlawi et al. (2022) used PageRank metric in their network-based predictor NetRank to detect robust cancer biomarkers and showed their method had a strong prediction performance.


(1) $${\mathrm{IIS}}_{i}=\sum_{j\in {\mathrm{NC}}_{i}}\frac{{\mathrm{PR}}_{j}}{d_{ij}}+\sum_{k\in C_{i}}\frac{{\mathrm{PR}}_{k}}{d_{ik}}\;\forall i\in C.$$

In Equation (1), C denotes the set of contact proteins of the cell in question. $C_{i}$ denotes the set of contact proteins to which contact protein i is directly or indirectly connected, and $NC_{i}$ denotes the set of intracellular proteins to which contact protein i is directly or indirectly connected in the cell. ${\mathrm{PR}}_{j}$ denotes PageRank centrality score of protein j, while $d_{ij}$ denotes the length of the shortest path between contact protein i and cell protein j in the cell PPI network.

**Figure 2.**
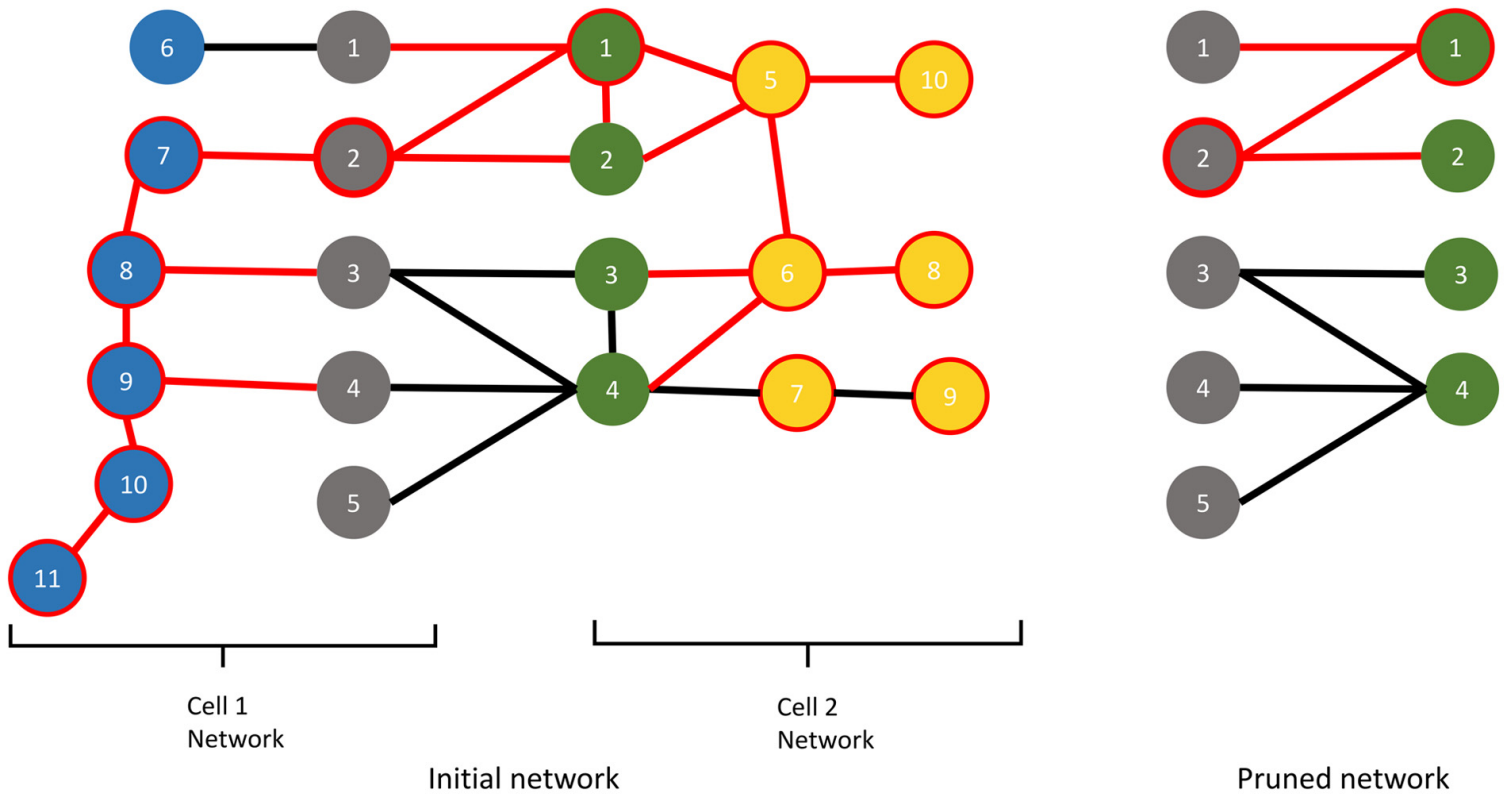
Small illustrations that show the initial and pruned PPI networks of two cells.

Since PPI networks have the small world phenomenon feature, which causes most of the proteins to have similar attributes, we developed a scoring method that can differentiate the scores of the proteins as much as possible. To this end, we calculated the IIS for each protein by evaluating the interactions between the contact proteins separately and using a distance metric to measure the scores more sensitively. We used the network by removing the edges between the contact proteins to obtain the distance parameters in the first component of Equation (1), and we only used the network of the contact proteins to obtain the distance parameters in the second component. Fig. 2 shows a small example of the initial and pruned PPI networks.

### 2.3 Finding the bridges

Assigning scores to the contact proteins considerably reduces the problem size, and yields a bipartite network where only contact proteins exist with node scores. Using integer linear programming (ILP), we determined the bridges on these bipartite networks.

We define the most significant protein pairs (i.e. bridges) as the minimum number of contact protein pairs that can cover a certain majority (specified using a parameter named *α*) of the cell-to-cell interactions and have the highest total IIS for the two intracellular networks. This problem is a variation of the ‘set cover with pairs problem’ (Hassin and Danny 2005). The main difference is that our aim is not necessarily to cover all elements (edges) by minimum costly pairs of objects (nodes/proteins) in our problem, while in the set cover with pairs problem the goal is to cover all elements by the selected pairs. The purpose of setting an *α* parameter instead of solving the set cover with pairs problem is to better prioritize the proteins, i.e. to find a smaller set of proteins to be focused on in the further biological research. Therefore our formulation becomes an extension to the maximum quasi-clique problem (Pattillo et al. 2013), which asks to find a subgraph with the edge density of *γ* ∈ (0, 1) in a graph *G*=(*V*, *E*). In addition, we select the pairs among only the connected pairs instead of all possible pairs of nodes. This selection method provides biologically more meaningful protein pairs, since each selected pair is an intercellular interacting pair meaning that they play a role in the cell-to-cell interaction of the two cells, and they may be involved in demyelination.

Our ILP model collectively evaluates the effect of the selected protein pairs on the network. It is also possible to evaluate the protein pairs separately. This can be done by sorting the nodes in descending order by the sum of the normalized degree of coverage (i.e. the number of edges a pair covers) and the normalized score of the pair. This sum is the ‘overall importance score (OIS)’ of a protein pair. We calculate the score of a pair by summing the normalized IIS of each protein in the pair.

#### 2.3.1 ILP model

We define P as the set of contact protein pairs (i.e. pairs of connected nodes) and E as the set of interactions (i.e. edges) in the network. Since we evaluate only the connected protein pairs, these two sets are equivalent in our implementation. We further define $c_{ij}$ as the binary parameter that indicates whether pair i covers interaction j. If one of the proteins in the pair is included in an interaction, it means that the pair covers that interaction. $s_{i}$ is the parameter that indicates the inverse of the sum (i.e. 1/sum) of the normalized IIS of each protein in pair i. Since this is a minimization problem where we want to find the minimum number of proteins with highest scores that cover the highest number of the edges, we assign a score to each pair which is equal to the inverse of the sum of the scores of the proteins in that pair. Finally, α parameter expresses the minimum desired coverage rate of the interactions in the network. When α=1, the problem becomes finding protein pairs that cover all of the interactions in the network (the set cover with pairs problem).

In the ILP model, we have two binary decision variables x and y. $x_{i}$ expresses if pair i is selected, while $y_{j}$ expresses if interaction j is covered by the selected protein pairs. Formulation of the problem is as follows:


$$\begin{array}{c} \min\sum_{i\in P}x_{i}s_{i}\\ \mathrm{subject}\;\mathrm{to}\\ \begin{array}{c} x_{i}c_{ij}\leq y_{j}\;\forall i\in P,\;j\in E\\ \sum_{j\in E}y_{j}\geq \alpha \left|E\right|\\ \begin{array}{c} y_{j}\leq \sum_{i\in P}x_{i}c_{ij}\;\forall j\in E\\ {x_{i},\;y}_{j}\in \left\{0,\;1\right\}\;\forall i\in P,\;j\in E. \end{array} \end{array} \end{array}$$

The first constraint ensures that an edge is covered if a pair covering it is selected. The second constraint provides that the number of the covered interactions satisfies the minimum desired coverage rate. Finally, the third constraint ensures an edge cannot be covered if a pair covering it is not selected. We solved the ILP using the Python Gurobi Solver (https://www.gurobi.com).

**Figure 3.**
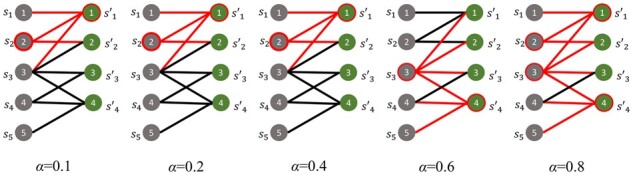
A small example of the selected proteins by BriFin based on different *α* values. Selected proteins and covered interactions are shown in red.

In Fig. 3, a small example of how selected contact protein pairs and covered interactions change based on the α parameter is shown.

### 2.4 Evaluating BriFin with MS patient data

#### 2.4.1 Subjects and isolation of PBMCs

We included eight RRMS patients and seven age and gender-matched healthy individuals as controls in this study. All patients are currently treated with immunomodulatory drugs. We presented the demographic and clinical features as well as immunomodulatory drugs of patients and healthy controls in Supplementary Table S1. After receiving written informed consent from all participants, we collected venous blood samples using tubes containing ethylene diamine tetra‐acetic acid. We separated PBMCs from venous blood via density gradient centrifugation using lymphocyte separation medium, density 1.077 g/ml (Capricorn). Finally, we pelleted the isolated PBMCs and stored them at −80°C for further use.

#### 2.4.2 Total RNA isolation and qRT-PCR

Total RNA was isolated from PBMCs with TriGent reagent (Biomatik) according to the manufacturer’s instructions. Quantification of RNA samples was conducted using the Nanodrop Spectrophotometer (ThermoFisher Scientific). RNA samples were reverse transcribed using the Advanced cDNA Synthesis Kit (Wisent Bioproducts) as recommended by the manufacturer. qRT-PCR was conducted on Bio Rad CFX Connect using The SensiFAST SYBR^®^ No-ROX Kit (Meridian Bioscience) with the primer pairs of Heat Shock Protein 90-alpha (HSP90AA1), HSP90B1, CALR, and Transferrin Receptor Protein 1 (TFRC) (for list of the primer sets, see Supplementary Table S2). The relative expression levels of each transcript were calculated by normalizing them against the expression of the housekeeping gene ß-actin (ACTB). For the fold change analysis, transcript levels were compared to the control group.

#### 2.4.3 Statistical analysis

We performed the statistical analysis of the results using GraphPad Prism version 8.0.0 (GraphPad Software, San Diego, California, USA). We used the non-parametric Mann–Whitney unpaired test to analyze the data, and we considered *P* < .05 to be statistically significant.

## 3 Results

### 3.1 Constructed networks

We ran BriFin on the networks that we constructed using PPI interaction data, cell-to-cell communication data, and proteome data downloaded from different sources as explained in Section 2. The numbers of all proteins and contact proteins (those involved in intercellular interactions) of each cell type in our study are given in Table 1.

**Table 1.** Sizes of the proteomes of each cell type used in this study.

Cell type	Number of the contact proteins	Total number of the proteins
Oligodendrocyte	282	2846
T-cell	647	8967
Macrophage	210	728

The first step of BriFin, network pruning, is run on the cell PPI networks shown in Table 1. The second step, ILP model, is run on the combined and pruned networks of two cells, oligodendrocyte–macrophage and oligodendrocyte–T-cell networks. The sizes of these networks are 2191 edges (i.e. contact protein pairs) for the oligodendrocyte–macrophage network, and 5983 edges for the oligodendrocyte–T-cell network.

### 3.2 Detected bridges by BriFin

After constructing the PPI networks summarized in Table 1, we calculated the IIS for each contact protein of the two cells. We reduced the intracellular network connections to these scores to be able to solve the problem efficiently, and obtained a bipartite network with node scores. We then assigned a score to each contact protein pair, and investigated the bridges among the contact protein pairs, each of which consists of one contact protein from Cell 1 and one contact protein from Cell 2, using the ILP model defined in Section 2.

To prioritize the contact protein pairs, we used different thresholds for α, which denotes the minimum ratio of cell-to-cell interactions covered by the selected contact protein pairs. Table 2 presents the number of the selected protein pairs for each setting of α for the two networks, we analyzed in this study.

**Table 2.** Number of the selected bridges by BriFin for different *α* values for the two networks.

Network\α	0.1	0.2	0.4	0.6	0.8
Oligodendrocyte-macrophage	2	5	11	21	36
Oligodendrocyte-T-cell	3	7	18	35	62

We identified that some of the selected proteins by the model have been associated with MS, other autoimmune diseases and neurological diseases in the literature. Here, we present the results for the two smallest α values: 0.1 and 0.2, based on the PageRank scores, and the relevant MS associations. For the oligodendrocyte–macrophage network, two bridges were selected by BriFin for α=0.1. These bridges are Integrin Beta-1 (ITGB1)—Heat Shock Protein 90-alpha (Heat Shock Protein 90-alpha) and HSP90AA1—TFRC, where the first protein belongs to oligodendrocyte and the second to macrophage. In the relevant literature, variants of *ITGB1* are found being associated with MS (Dardiotis et al. 2019). In a study where MS-specific membrane-associated biomarkers were investigated in experimental autoimmune encephalomyelitis (EAE), an animal model of MS, it was shown that TFRC protein expression was down-regulated in PBMCs (Dagley et al. 2014). In our study, we experimentally tested TFRC in blood samples of MS patients, and found that its mRNA expression is decreased in MS patients compared to healthy individuals. It is also important to observe that HSP90AA1 is important for both the immune cells and the oligodendrocyte cell. In a transcriptomic study (Schirmer et al. 2019), the up-regulation of *HSP90AA1* was shown in myelinating oligodendrocytes at MS periplaque white matter tissue. We also show in this study its association with MS on PBMCs of a group of MS patients. To the best of our knowledge, a PBMC association of HSP90AA1 with MS was not shown in the literature.

For the same network, five bridges were selected by BriFin for α=0.2, which are High Mobility Group Box 1 (HMGB1)—Vascular Cell Adhesion Molecule 1 (VCAM1), HSP90AA1—TFRC, ITGB1— Annexin A2 (ANXA2), Fibronectin 1 (FN1)—Pyruvate Kinase M1/2 (PKM), and Amyloid Beta Precursor Protein (APP)—HSP90AA1. We see that some proteins are again selected for the higher α value either in the same or in different bridges. All of these selected proteins are associated with MS in the literature in studies focusing on different mechanisms and cells.

Regarding associations of these proteins related to the cells belong to in our study, it was shown that HMGB1 expression levels were increased in PBMCs of MS patients significantly (Malhotra et al. 2015, Paudel et al. 2019), and various VCAM1 positive microglia/macrophages exist at the edges of MS lesions (Peterson et al. 2002). Also, *VCAM1* and its variants are associated with MS in several studies (Dardiotis et al. 2019). That APP plays a role in MS was shown in several studies (Gehrmann et al. 1995, Matías-Guiu et al. 2016). Gehrmann et al. (1995) showed that the level of APP expression is correlated with histopathological lesion development; therefore, APP is an important biomarker for the progression of MS. Also, Matías-Guiu et al. (2016) stated APP has a role in both demyelination and remyelination. Variants of *FN1* are shown to be associated with MS in the study of Dardiotis et al. (2019).

For the oligodendrocyte–T-cell network, three bridges are selected by BriFin for α=0.1. These are APP—APP, HSP90AA1—Epidermal-Growth Factor Receptor (EGFR), and FN1—ITGB1 in the respective order of the cells. There are some proteins selected in common with the oligodendrocyte–macrophage network, such as APP, HSP90AA1, FN1, and ITGB1. Scalabrino (2021) stated that recent findings show that EGF expression was significantly decreased in the cerebrospinal fluid (CSF) and SC of the MS patients, and the new information about the role of EGF in MS required a critical reassessment of the MS pathogenesis.

**Figure 4.**
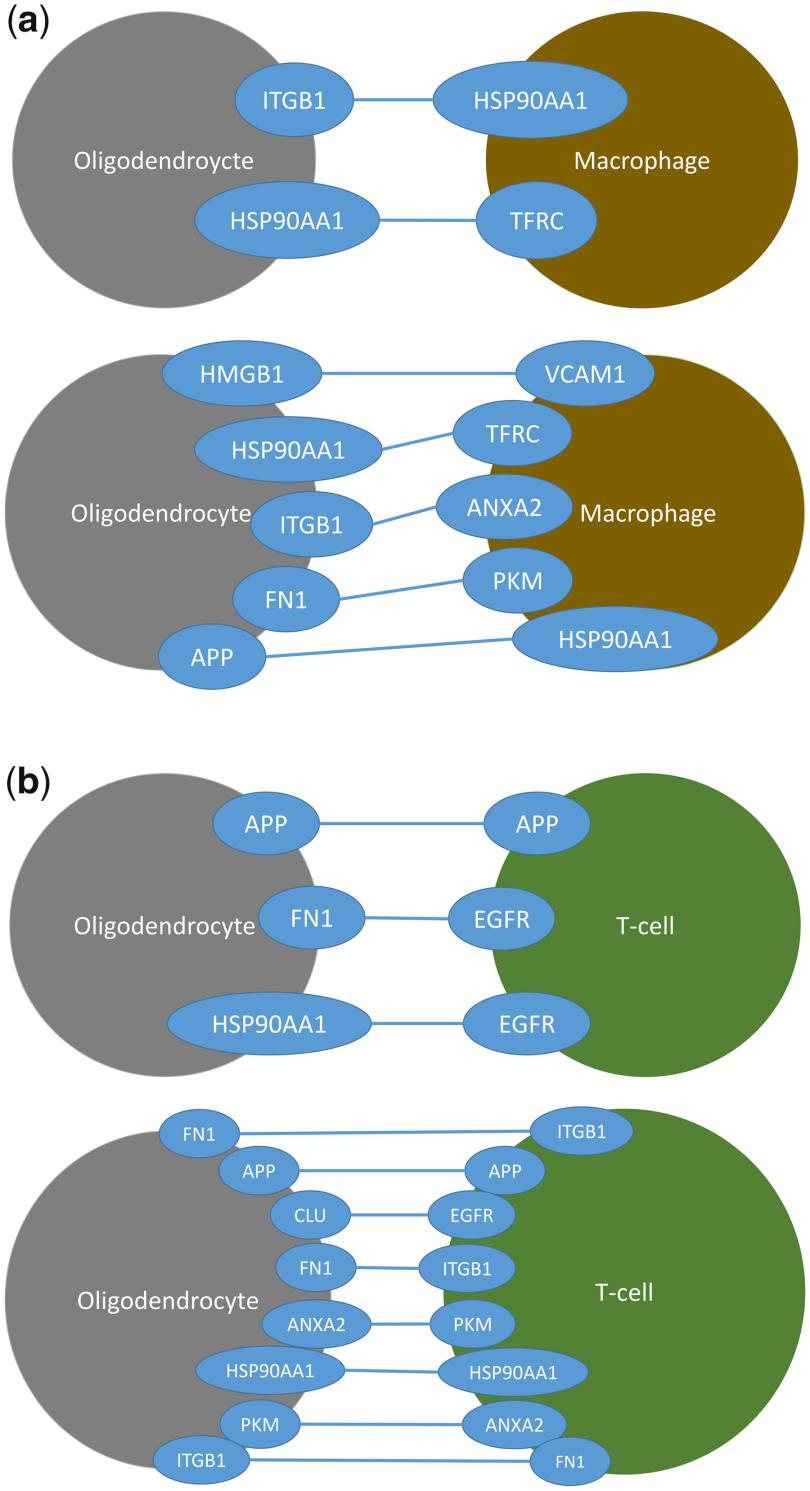
Visualizations of the selected PPI bridges for (a) the oligodendrocyte–macrophage network for α = 0.1 (top) and α = 0.2 (bottom), and for (b) the oligodendrocyte–T-cell network for α = 0.1 (top) and α = 0.2 (bottom).

For the same network, seven bridges are detected by BriFin α=0.2: Clusterin (CLU)—EGFR, PKM—ANXA2, ANXA2—PKM, ITGB1—FN1, HSP90AA1—HSP90AA1, APP—APP, and FN1-ITGB1. That the similar proteins are detected and there are bridges consisting of the same protein is worth mentioning. While PKM was not associated with macrophages in the relevant literature, a study based on an EAE model (Seki et al. 2020) emphasizes the therapeutic potential of PKM2 activators in MS-like diseases since they change T-cell function, and shows how these activators change T-cell function. In Fig. 4, the selected bridges for α= 0.1 and α= 0.2 values for the two networks are visualized.

For larger α values, there are also MS-associated proteins, such as CD44 Antigen (CD44), Apolipoprotein E (APOE), Aldolase A (ALDOA), Interleukin-7 Receptor (IL7R), and Major Histocompatibility Complex, Class II, DR Beta 1 (HLA-DRB1) among the selected proteins by BriFin. In the domain of MS research, there are many studies that demonstrate the role of IL7R for the disease. For instance, Lei et al. (2017) showed that IL7R is down-regulated during demyelination, and by targeted knockdown experiments, they also showed that IL7R is crucial for myelination in embryonic and larval zebrafish. Barcellos et al. (2006) reported that there is a strong association between certain variants of *HLA-DRB1* and MS in a comprehensive study that includes data from diverse populations. Farias et al. (2014) reported that *ALDOA* and *APOE* genes are up-regulated in CSF of MS patients. In addition, Guan et al. (2011) showed that CD44 controls the development of EAE.

Selected bridges where both involved proteins are MS-associated may provide useful information about the disease mechanism. Thus, we mention some of these bridges, where the first protein belongs to oligodendrocyte, the second to immune cell: APOE—VCAM1, HLA-DRB1—PKM, APOE—ALDOA, and FN1—CD44.

The full lists of the selected proteins for all α values are given in Supplementary Tables S3–S12. We also report the selected protein pairs when the Betweenness Centrality scores are used as an alternative to the PageRank scores to calculate the IIS for each protein, along with the betweenness-based OIS for each protein pair since it is a commonly used metric. This analysis yields similar results to the PageRank-based analysis with some changes in the prioritization order or in the proteins. Similarly, we give the complete lists of the betweenness-based results in Supplementary Tables S13–S22, and we show top 10 highest-scoring (in terms of OIS) bridges among the selected ones by BriFin for all tested values in Table 3.

**Table 3.** Top 10 bridges selected by BriFin for all tested *α* values sorted by highest OIS (proteins in respective order).

Α	Pair in oligodendrocyte–macrophage network		α	Pair in oligodendrocyte–T-cell network	
0.2	APP	HSP90AA1	0.1	FN1	ITGB1
0.2	FN1	PKM	0.1	APP	APP
0.1	HSP90AA1	TFRC	0.4	FN1	ITGAV
0.1	ITGB1	HSP90AA1	0.1	HSP90AA1	EGFR
0.2	ITGB1	ANXA2	0.8	FN1	ITGA4
0.4	PKM	HSP90AA1	0.2	ITGB1	FN1
0.4	FN1	CD44	0.4	APP	TFRC
0.4	HSP90AA1	CTSD	0.2	HSP90AA1	HSP90AA1
0.4	APP	CALR	0.4	ITGB1	C1QBP
0.4	CTSD	PKM	0.2	CLU	EGFR

It is important to note that, due to the protein pairs that have similar scores, many alternative optimal solutions exist for some α values. However, these solutions include mostly the same proteins. In addition, the BriFin model evaluates the collective effect of the protein pairs on the network, which means that it chooses the highest-scoring protein pairs whose interactions are complementary. We list the individual scores for the protein pairs (OIS) in Supplementary Tables S23–S26. Table 4 shows the top 10 bridges for the two networks according to this scoring metric (with PageRank-based scores).

**Table 4.** Top 10 bridges for each network based on the OIS (proteins in respective order).

Order	Pair in oligodendrocyte–macrophage network		Pair in oligodendrocyte–T-cell network	
1	APP	PKM	FN1	EGFR
2	APP	HSP90AA1	FN1	APP
3	HSP90AA1	PKM	FN1	HSP90AA1
4	HSP90AA1	HSP90AA1	FN1	FN1
5	FN1	PKM	FN1	ITGB1
6	FN1	HSP90AA1	FN1	PKM
7	APP	TFRC	FN1	TFRC
8	HSP90AA1	TFRC	FN1	ANXA2
9	FN1	TFRC	APP	EGFR
10	APP	ANXA2	FN1	ALDOA

In our analysis, we also identified the top contributors to the scores of the selected contact proteins among the intracellular proteins to prioritize the intracellular proteins. Proteins connected with shorter paths on the network are likely to contribute more because of the formula we used to calculate the IIS. However, individual PageRank scores are also effective on the IIS. That is, the top contributors for a contact protein may be interpreted as the ones having the highest centrality scores among the proteins close to it on the network. For each cell, we identified the top 20 score contributors for the selected highest scoring 10 proteins. We show the most frequent intracellular contributors among the top 20 in Supplementary Table S27, for each cell.

### 3.3 Experimental validation of several proteins detected by BriFin

**Figure 5.**
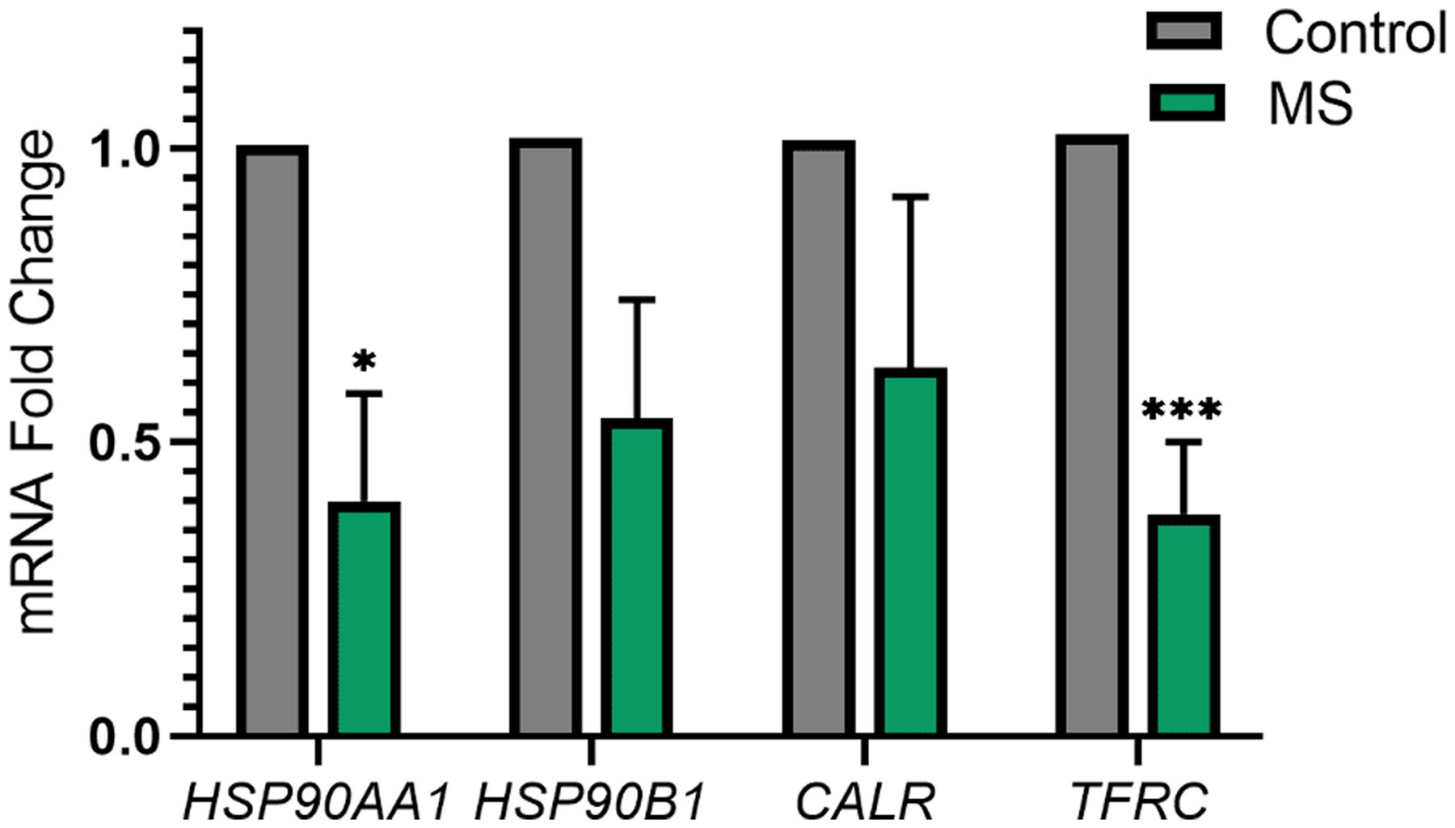
HSP90AA1, HSP90B1, CALR, and TFRC mRNA expressions in PBMCs of control and MS patients. Selected genes were analyzed by qRT-PCR. Fold changes in expression levels of MS patients (*n* = 8, green bars) compared with the healthy controls (*n* = 7, gray bars) were shown in the graph bar. qRT-PCR analysis indicates that the mRNA levels of the HSP90AA1 and TFRC were significantly low in the MS group relative to healthy controls. The error bars are presented as means ± SEM, *P*-values; *<0.05, ***<0.001, Mann–Whitney *U*-test.

To experimentally validate our predictions, we selected four proteins among the proteins in the highest-scoring pairs that likely play a role in demyelination and investigated their mRNA expression levels in the blood samples obtained from MS patients. These proteins are HSP90AA1, HSP90B1, CALR, and TFRC. The expression levels of the selected proteins’ transcripts in PBMCs were determined by qRT-PCR. Our results showed that the expression levels of the HSP90AA1, and TFRC reduced significantly in the MS group when compared to the control group (Fig. 5).

### 3.4 Statistical evaluation

To statistically evaluate the performance of BriFin, we compared BriFin results with random selection results. Since there is no disease-association database that includes specific information about the associations, such as cell types and mechanisms for MS, we conducted a literature search for the distinct contact proteins in the oligodendrocyte–macrophage network, which is relatively smaller. We investigated 392 distinct proteins, involved in the oligodendrocyte–macrophage contact, through evaluating the first 5–10 PubMed articles for each protein, and detected the MS-associations based on the cell type where the association was shown. We also added the associations we experimentally validated. For some proteins, we found no studies that include associations regarding the protein–cell combinations. Therefore, for these cases, we determined associations based on studies that show the association on analyzed EAE and MS samples, such as PBMC, serum, plasma, CSF, brain, and SC lesions, or show the association for some variants of the protein. In these cases, if the association has the potential to be related to the cell in question, we also considered that protein to be associated with MS.

There exist 232 contact proteins for oligodendrocyte and 208 contact proteins for macrophage that are involved in the intercellular interactions in the oligodendrocyte–macrophage PPI network. We identified that 82 of the oligodendrocyte contact proteins and 124 of the macrophage contact proteins are associated with MS in studies that are related to the cell they belong to in our study.

There are also several studies that list the other proteins as associated with MS, which we accepted as non-associated for oligodendrocytes and macrophages, since they were reported in experiments that use other types of cells, such as astrocyte, T-cell, B-cell, and microglia. Since we evaluated the predictions of important actors in the oligodendrocyte–macrophage network, we ignored them to provide cell-type-specific statistics. Complete lists of the proteins associated with MS and the related references used in this study are given in Supplementary Tables S28 and S29 for oligodendrocyte and macrophage proteins, respectively.

To measure the performance of BriFin based on the *α* values, we randomly selected as many proteins as the number of contact protein pairs selected by BriFin for each *α* value among the contact protein pairs that we determined for the oligodendrocyte–macrophage network. We repeated this random selection 10 000 times. Based on the MS-associated proteins, we found by our literature search, we calculated the rate of MS-associated proteins among the selected proteins by BriFin and random selection. In Table 5, we compare the rate of MS-associated proteins detected by BriFin with the average rates of the MS-associated proteins selected randomly for each α value.

**Table 5.** Performance of BriFin compared to random selection for different α values.

α	Random selection—average rate of disease associated proteins among the selected ones (%)	BriFin—rate of disease associated proteins among the selected ones (%)
0.1	58	100
0.2	57	80
0.4	55	64
0.6	52	62
0.8	48	61

As seen in Table 5, BriFin detects more MS-associated proteins from the contact protein pairs for all α values. Random selection has a similar rate of MS-associated proteins for all α values, while BriFin has a higher rate of MS-associated proteins for lower α values, which supports our assumption that the lower α values provide protein bridges with higher importance.

### 3.5 BriFin run time

BriFin consists of a network pruning heuristic in which the IIS score is calculated for each contact protein of the two cells, and an ILP model to detect the bridges on the reduced network with contact protein pair scores. When α=1, our bridge finding problem is a derivative of the set cover with pairs problem, which is NP-Complete [36]. For lower α values, the bridge finding problem becomes a derivative of the quasi-clique problem, which is also NP-Complete [37]. Because of the network pruning heuristic of BriFin, which simplifies the graph into a bipartite graph, the run time becomes feasible for the ILP problem. To demonstrate the network pruning run time, we randomly subsampled the T-cell network based on the number of edges (i.e. interactions). We present the network pruning run times for the T-cell case based on the network size in terms of the edges in Table 6.

**Table 6.** Run time of the network pruning heuristic for the T-cell proteome based on the number of edges.

Number of edges	Run time (s)
29 423	3003.17
58 846	11 810.75
117 691	55 569.09

To demonstrate the ILP run time, we ran the ILP model on the oligodendrocyte–T-cell network using different α values. Additionally, we subsampled the network to analyze the ILP run time with respect to the network size, by halving the number of protein pairs and setting α to 0.4. We observe that the ILP run time scales with *α* values, as well as the network size. We provide the run time analysis for the ILP problem in Tables 7 and 8. As shown in Tables 6 and 7, the cell network pruning heuristic takes considerably more time than the ILP problem.

**Table 7.** Run time of the BriFin ILP model based on the value of α.

α	Run time (s)
0.1	177.04
0.2	213.4
0.4	1685.68
0.6	1292.92
0.8	2982.23

**Table 8.** Run time of the BriFin ILP model based on the network size.

Number of contact protein pairs	Time (s)
748	0.9
1496	11.65
2992	174.22
5983	1685.68

## 4 Discussion

Network science is an essential tool to infer physiological interpretation from biological networks since it evaluates the networks with a holistic view, and also a good way to support biological studies since the resources are limited and there is a great deal of relevant data to eliminate and prioritize. Here, we presented the BriFin model to detect bridges, key protein–protein pairs, between oligodendrocytes and macrophages or T-cells. We showed that the detected proteins by our model were associated with MS, and two detected proteins were differentially expressed in MS patients in an application of network analysis. That the hubs detected by the model are also important proteins to investigate because of the biological mechanisms they are involved in is a meaningful result, and proteins/genes that are both biologically and mathematically pointed out might be good starting points to do more research on.

There are many proteins that are associated with MS in the protein group that we grouped as “contact proteins.” However, this information makes it harder to choose a research target. Most probably, some proteins are the causes that originate the demyelination problem and the others are the consequences of them. So prioritization, even among disease-associated proteins, is important, and our aim was to prioritize the proteins and present important information that could lead to the exploration of new disease proteins.

Among the selected protein pairs for all the tested *α* values, there are pairs whose both proteins are MS-associated. Investigating the biological mechanisms behind the interaction of the proteins in these pairs may yield useful information to understand MS better. In addition, proteins that are the matches of the proteins associated with MS are good research targets for further studies. Also, research on selected proteins that are associated with autoimmune diseases and other neurodegenerative diseases may yield useful information about MS. Finally, the selected proteins and the highest-scoring pairs that have not been associated with any disease yet might be potential research directions.

The provided results are based on the collected interaction and proteome data. Therefore, the quality of the results depends on the quantity and the quality of the data. These computational results can become more reliable and quality by more data and more biological expertise. The distance of the biological assumptions from reality and the level of the inclusion of these assumptions are important for computational studies, and improvements on these topics might be new research directions.

Our network analysis approach might be useful for other diseases where two cell types interact, such as autoimmune diseases, cancer, many neurological diseases, and for research areas in which cell-to-cell interactions are dominant, such as immunotherapy and microbiome–host interaction.

## Supplementary Material

btad175_Supplementary_Data

## Data Availability

The data underlying this article are available in the article’s online supplementary material. The time we downloaded data is 19 August 2021 for IntAct, and 2 November 2021 for BaderLab Cell–Cell Interaction Database and CellTalkDB.
